# Preparation and Characterization of Ternary Complexes to Improve the Solubility and Dissolution Performance of a Proteolysis-Targeting Chimera Drug

**DOI:** 10.3390/pharmaceutics17050671

**Published:** 2025-05-20

**Authors:** Heng Zhang, Hengqian Wu, Lili Wang, Laura Machín Galarza, Chuanyu Wu, Mingzhong Li, Zhengping Wang, Erpeng Zhou, Jun Han

**Affiliations:** 1 School of Chemistry and Chemical Engineering, University of Jinan, Jinan 250022, China; hengzhangmail@163.com; 2 Institute of Biopharmaceutical Research, Liaocheng University, Liaocheng 252000, China; 3 Institute of Pharmacy and Foods, University of Havana, Havana 17100, Cuba; 4 School of Chemistry and Chemical Engineering, University of Surrey, Guildford GU2 7XH, UK; 5 School of Pharmacy, De Montfort University, Leicester LE1 9BH, UK; 6 Liaocheng High-Tech Biotechnology Co., Ltd., Liaocheng 252000, China; 7 College of Chemical Engineering, Shijiazhuang University, Hebei International Joint Research Center for Biopharmaceutical, Shijiazhuang 050035, China

**Keywords:** proteolysis targeting chimeras (PROTACs), sulfobutyl ether-β-cyclodextrin, vitamin E-TPGS, ternary complex, dissolution enhancement

## Abstract

**Background/Objectives:** Proteolysis-targeting chimeras (PROTACs) have shown significant potential in the treatment of intractable diseases. However, their clinical applications are limited by poor water solubility and permeability. In this study, the cyclodextrin inclusion method was employed for the first time to prepare the PROTAC-CD complex with the aim of improving the dissolution of a PROTAC drug (LC001). **Methods:** Initially, sulfobutyl ether-β-cyclodextrin (SBE-β-CD) was selected to improve the solubility of LC001. The polymer TPGS was screened based on the phase solubility method to enhance the efficiency of complexation and solubilization capacity, and its ratio with SBE-β-CD was optimized. The ternary complex was prepared by lyophilization with an SBE-β-CD/TPGS molar ratio of 1:0.03. Differential scanning calorimetry, powder X-ray diffraction, and scanning electron microscopy results confirmed the formation of an amorphous complex. Fourier-transform infrared and molecular docking simulations indicated the formation of hydrogen bond interactions between components. **Results:** The results showed that the ternary complexes significantly improved the dissolution rate and release amount of LC001 in PBS (pH 6.8) and were unaffected by changes in gastric pH compared to the binary complexes and physical mixtures. The lack of crystal structure in the lyophilized particles and the formation of nano aggregates in solution may be the reasons for the improved dissolution of the ternary complex. **Conclusions:** In conclusion, the addition of TPGS to the LC001-SBE-β-CD binary system has a synergistic effect on improving the solubility and dissolution of LC001. This ternary complex is a promising formulation for enhancing the dissolution of LC001.

## 1. Introduction

Proteolysis-targeting chimeras (PROTACs) are emerging as therapeutic modalities for disease treatment. By hijacking the endogenous ubiquitin–proteinase system, PROTACs can achieve therapeutic efficacy by selectively inducing the degradation of target proteins at low doses in a long-circulating manner [1]. Compared to traditional small-molecule inhibitors, PROTACs can overcome drug resistance, reduce toxic side effects, and address undruggable targets, making them indispensable in treating intractable diseases [2], particularly cancer [3]. To date, more than 25 small-molecule PROTACs have entered clinical research [4].

Despite their outstanding properties for disease treatment, PROTACs have some drawbacks. PROTAC molecules exceed Lipinski’s Rule of Five (Ro5) because of their unique ternary structures [5]. Some factors are unfavorable for oral absorption, such as low solubility, poor permeability, and unpredictable hook effect, hindering the clinical translation of PROTACs [6]. To accelerate the transition of PROTAC drugs from the laboratory to clinical practice, researchers have developed various delivery systems, such as nanoparticles [7,8], polymer micelles [9], microneedle patches [10], composite hydrogels [11], and nucleic acid-based [12] delivery systems. Although lacking oral drug properties, most PROTACs are administered orally at the clinical stage [13]. Oral administration remains the preferred delivery method for PROTACs because of its convenience, stability, high patient compliance, and cost-effectiveness [14]. Currently, only a limited number of articles have reported the use of amorphous solid dispersion (ASD) technology in preparing oral solid formulations of PROTAC [15,16]. Although the prepared ASD formulation increased and maintained the supersaturation of PROTAC, the dissolution rate and degree were low, and the dissolution performance decreased under high drug loading.

Cyclodextrin inclusion is an established method for preparing amorphous solid dispersions, that enhance the solubility of poorly water-soluble drugs [17]. However, the limited water solubility of natural cyclodextrins (CDs) can lead to drug precipitation. To overcome this challenge, sulfobutyl groups replace the hydrogen atoms of the hydroxyl groups in CDs, forming sulfobutyl ether-β-cyclodextrin (SBE-β-CD) [18]. Compared to the parent β-CD, SBE-β-CD exhibits higher water solubility, lower toxicity, and enhanced drug complexation capabilities. SBE-β-CD has been extensively explored for the formulation of inclusion complexes (ICs) to improve the solubility and dissolution properties of hydrophobic compounds [19], mitigate fast-fed variability, protect the stability of sensitive compounds, and reduce interference from the biological environment during drug absorption, distribution, and metabolism [20]. In addition, SBE-β-CD forms ICs with insoluble and poorly permeable drugs, enhancing their permeability through the paracellular transport pathway of biological membrane water channels [21]. SBE-β-CD can also act as a permeation enhancer by altering the integrity of the epithelial membrane, improving the bioavailability of insoluble drugs [22]. Formulations based on SBE-β-CD ICs have been administrated by various routes, providing an intelligent solution for the clinical translation of poorly soluble drugs. More than 15 products utilizing SBE-β-CD have been approved for commercialization by the FDA [22].

Although CDs offer the advantage of improved solubility, high concentrations of CDs are required to demonstrate this benefit. Additionally, excessive amounts of CDs increase the formulation volume, exceeding the acceptable range for oral administration, and making it less favorable for oral delivery [23]. Therefore, to enhance the complexation efficiency (CE) of cyclodextrins with drug molecules, researchers have formulated ternary complexes by adding hydrophilic polymers [24], such as PEG, HPMC, Soluplus, PVP, chitosan, and poloxamer407. These polymers form stable complexes with CDs or drug molecules through van der Waals forces, hydrophobic interactions, ion–ion interactions, electrostatic interactions, and hydrogen bonding [24]. Studies have shown that these excipients improve the dissolution rate and bioavailability of drug molecules by enhancing particle wettability and forming easily soluble complexes [25]. Ternary agents used to form multi-component systems help to improve solubility and reduce the required amount of CD. Furthermore, multi-component systems can be formulated in any dosage form to achieve the desired therapeutic effect [22]. Thus, ternary cyclodextrin systems represent a promising approach to enhance the solubility of poorly soluble drugs.

The present study aimed to screen and optimize cyclodextrin inclusion complexes to enhance the solubility and dissolution rate of LC001 in a simulated intestinal environment.

The structure of LC001 was similar to that of ARV-471, as shown in Figure S1. As an innovative PROTAC drug, the fundamental chemical properties and preliminary druggability evaluation of LC001 could be found in the patent [26]. We also focused on avoiding the impact of gastric pH variability on the dissolution of LC001. The LC001/SBE-β-CD/polymer ternary complex was prepared using the freeze-drying method. In vitro dissolution properties and physical stability of ternary complex were evaluated. The result demonstrated that the ternary complex serves as an ideal drug delivery system for improving the solubility and dissolution performance of PROTAC drugs.

## 2. Materials and Methods

### 2.1. Materials

The PROTAC drug LC001 was provided by the Haisco Pharmaceutical Group (Sichuan, China). The chemical structure of LC001 is presented in Figure S1. Eudragit L100 (L100) was obtained from Evonik (Kirschenallee, Germany). Hypromellose acetate succinate (HPMCAS-LG) was provided by Ashland (Louisville, KY, USA). Polyvinylpyrrolidone (Kollidon^®^30, PVP), polyethylene-polypropylene glycol (Poloxamer 407), and polyvinyl caprolactam-polyvinyl acetate-polyethylene glycol (Soluplus) were purchased from BASF (Ludwigshafen, Germany). D-α-tocopheryl-polyethyleneglycol-1000-succinate (TPGS) was obtained from Ron Pharmaceutical Technology Co., Ltd. (Shanghai, China). Sodium dihydrogen phosphate (NaH_2_PO_4_, AR grade) and sodium hydroxide (NaOH, AR grade) were gained from Sinopharm Chemical Reagent Co., Ltd. (Shanghai, China). Dimethyl sulfoxide (DMSO, HPLC grade) and acetonitrile (HPLC grade) were purchased from Adamas Pharmaceuticals, Inc. (Shanghai, China). All aqueous solutions were purified by a Milli-Q^®^ water system (Millipore, Bedford, MA, USA) in the laboratory.

### 2.2. Phase Solubility Study

The phase solubility study was conducted by employing a previously reported method [27]. To compare the solubilizing effects of HP-β-CD and SBE-β-CD on LC001, phase solubility studies were performed using cyclodextrin solutions at concentrations of 5–100 mmol/L. Solutions of HP-β-CD and SBE-β-CD at concentrations of 5, 10, 20, 40, 60, 80, and 100 mM were prepared in both water and phosphate buffer (pH 6.8). In addition, aqueous solutions of SBE-β-CD at different concentrations containing 10% (*w*/*w*) TPGS and Soluplus polymers were prepared to investigate the effect of additives on solubilization. An excess of LC001 powder was added to the aforementioned solution, which was sonicated for 30 min and shaken for 48 h at 37 °C. After equilibration, the sample solution was filtered through a 0.45 µm membrane filter and analyzed using UV-Vis spectrophotometry at 263 nm (S3100 UV-Vis spectrophotometer, SCINCO, Gasan-dong, Deung-gu, Daejeon, Seoul, Republic of Korea). The concentration of dissolved LC001 was determined using the external standard method. All experiments were performed in triplicates. Phase solubility profiles were constructed by plotting the concentration of dissolved LC001 (mmol/L) against the cyclodextrin concentration (mmol/L). In addition, the complexation constants (*K*_S_) and complexation efficiencies (CE) of the binary LC001-HP-β-CD, LC001-SBE-β-CD, and ternary LC001-SBE-β-CD-polymer systems were evaluated. The *K*_S_ and CE were calculated according to Equations (1) and (2) [28].

(1) $$K_{S}=\frac{\mathrm{slope}}{S_{0}\left(1-\mathrm{slope}\right)}$$

(2) $$\mathrm{CE}=\frac{\mathrm{slope}}{1-\mathrm{slope}}$$

The slope was quantified by fitting the phase solubility curve, and *S*_0_ represents the solubility of LC001 (mmol/L) in pure aqueous solution (the measured *S*_0_ for LC001 is 0.05 mmol/L). In the saturated drug aqueous medium, the drug–cyclodextrin molar ratio (D:CD) was calculated using the formula:

$$D:\mathrm{CD}=1:\frac{1+\mathrm{CE}}{\mathrm{CE}}$$

### 2.3. Molecular Docking Simulation

Molecular docking simulations were conducted using software AutoDock Vina 1.2.5, which employs a sophisticated gradient-based local optimization algorithm [29]. The molecular structure of LC001 was generated using the ChemDraw 2014 software. The molecule was energy-minimized using the MMOL/LFF94 force field in Chem3D 2014 software [30,31] to obtain the most stable molecular conformation, saved in PDB format. The molecular structures of SBE-β-CD (PubChem CID:131634907) and HP-β-CD (PubChem CID:131634907) in SDF format were obtained from the PDB database (website: https://pubchem.ncbi.nlm.nih.gov/, accessed on 1 December 2024). Because the average degrees of substitution for SBE-β-CD and HP-β-CD used in the experiments were 6.5 and 4.5, respectively, Chem3D 2014 software was employed to remove or add substituents in the CD molecules to obtain 6-substituted and 7-substituted SBE-β-CD, as well as 4-substituted and 5-substituted HP-β-CD molecular structures [22]. The MMOL/LFF94 force field was used to optimize the energy of the molecules to obtain stable molecular conformations, saved as PDB format files. The Auto Dock Tools 1.5.6 program was used to preprocess cyclodextrin and the LC001 molecule, saved in pdbqt format. Cyclodextrin was treated as rigid during the docking process, whereas the LC001 molecule was designated as flexible. The box size in the configuration file was set to fully encompass cyclodextrin and the LC001 molecule. The exhaustiveness value was set to ten. Vina determined the best scoring mode from the docking results, and the analysis was performed using the PyMOL software (website: http://www.pymol.org/, accessed on 5 December 2024).

### 2.4. Screening of Precipitation Inhibitors

#### 2.4.1. Saturated Solubility of LC001 in Polymer Solutions of Different Concentrations

The solubility of LC001 was determined at different concentrations of hydrophilic polymers. Phosphate buffer solutions (PBS, pH 6.8) with varying polymer concentrations (1, 2, 5, and 10 mg/mL) were prepared separately. An excess of LC001 powder was added to these solutions. After shaking at 37 °C and 175 rpm for 48 h, the mixtures were centrifuged, and the supernatant was collected. An equal volume of acetonitrile was added to prevent the precipitation of the API. The concentration of LC001 was quantified at 263 nm using a UV-Vis spectrophotometer (S3100, SCINCO, Gasan-dong, Deung-gu, Daejeon Seoul, South Korea). The absorbance was corrected by subtracting the blank control solution. All tests were performed in triplicate.

#### 2.4.2. Precipitation Inhibition Study

The solvent transfer method [32] was employed to investigate the effects of different concentrations of TPGS, SBE-β-CD, and their mixed solutions on the supersaturation of LC001. PBS (pH 6.8) containing TPGS at concentrations of 0.3, 1.0, and 1.5 mg/mL and SBE-β-CD at concentrations of 1.5, 5.0, and 10.0 mg/mL were prepared. Additionally, the mixed solutions of 1.5 mg/mL SBE-β-CD with 0.3, 1.0, and 1.5 mg/mL of TPGS were prepared separately. The concentrations of SBE-β-CD and TPGS in the mixed solution corresponded to the concentrations of F2, F3, and F4 dissolved in 100 mL of the medium. Subsequently, 1 mL of LC001-DMSO stock solution at a concentration of 25 mg/mL was added to 20 mL of PBS (pH 6.8) containing varying concentrations of SBE-β-CD and TPGS. The mixed solution was stirred (150 rpm) at 37 °C for 180 min. At scheduled time intervals, 1 mL of the solution was collected and dissolved in the same volume of acetonitrile. Simultaneously, the same volume of fresh medium was replenished into the dissolution cup. A pure PBS medium without polymer was prepared concurrently to eliminate the influence of organic solvent. The dissolution samples were obtained using the same operational method. The LC001 concentration was quantified as described in Section 2.4.1. All tests were performed in triplicate.

#### 2.4.3. Saturated Solubility of LC001 in Solutions with Different SBE-β-CD/TPGS Molar Ratios

The saturated solubility of LC001 was investigated in solutions with SBE-β-CD (C_SBE-β-CD_ = 50 mmol/L)/TPGS molar ratios of 0:0, 0:0.01, 0:0.03, 0:0.05, 1:0.01, 1:0.03, and 1:0.05. Solutions with different SBE-β-CD/TPGS molar ratios were prepared using an aqueous medium and stirred until clear. An excess of LC001 powder was added to the solutions, which were sonicated for 30 min and then stirred in a 45 °C water bath for 48 h. After reaching equilibrium, the sample solutions were filtered through a 0.45 µm membrane filter and analyzed using UV spectrophotometry at 263 nm (S3100 UV-Vis spectrophotometer; SCINCO, Gasan-dong, Deung-gu, Daejeon, Seoul, South Korea). The concentration of dissolved LC001 was determined using the external standard method. All samples were tested in triplicate.

### 2.5. Particle Size and Zeta Potential Measurement

The particle size and zeta potential of the test solution were studied using the Malvern Nano ZSP instrument (Malvern Instruments, Malvern, UK). The testing temperature was 25 °C, with an equilibrium time of 120 s and a measurement angle of 173°. The instrument was calibrated using Nanosphere™ size standards (Thermo Fisher Scientific, Newington, CT, USA). Before testing, the sample solution was filtered through a 10-micrometer polyethylene filter to remove undissolved solid particles.

### 2.6. Polarized Light Microscope (PLM) Observation

The images were obtained with a polarized light microscope (Axioscope, Zeiss, Oberkochen, Germany). PLM was used to visualize the morphology and birefringence pattern of samples (pure LC001, binary and ternary complex).

### 2.7. Preparation of the Inclusion Complexes

The binary and ternary inclusion complexes were prepared using the freeze-drying method [17,33]. In order to compare the differences in dissolution of ternary complexes, 5%, 10%, and 20% (*w*/*w*) of TPGS and Soluplus were added to the binary LC001-SBE-β-CD system for preparing the ternary complexes. Additionally, to screen the dosage of TPGS, the ternary complex formulations were prepared according to the component ratios in Table 1.

Polymers (TPGS and Soluplus) and SBE-β-CD (50 mmol/L) were added to an aqueous solution and stirred to dissolve. The LC001 powder was added according to the pre-determined molar ratio. After sonication for 30 min, the solution was stirred in a water bath at 45 °C for 24 h and passed through a 0.45 µm membrane filter. The filtered solution was transferred to a Petri dish and frozen in an ultra-low temperature freezer (MDF U53V, Panasonic, Osaka, Japan) at −80 °C for two hours. The pre-frozen solids were freeze-dried using the lyophilizer (Benchtop pro31, SP Scientific, New York, NY, USA) under a vacuum at −50 °C for 24 h. The lyophilized samples were sieved through a 60-mesh screen and stored in a sealed desiccator for further study.

### 2.8. Characterization of Complexes

#### 2.8.1. Thermal Analysis

The thermogravimetric (TGA) and differential scanning calorimetry (DSC) analyses were conducted with a thermogravimetric analyzer and differential scanning calorimeters (Discovery, TA Instruments, New Castle, DE, USA). For the TGA test, 4–8 mg of the sample was accurately weighed into an aluminum pan without a lid. To determine the melting point of LC001, 2–4 mg of LC001 powder was accurately weighed into a pinhole aluminum pan. The sample was heated from 20 °C to 250 °C at 10 °C/min under a standard measurement mode. The furnace was purged with nitrogen gas at a flow rate of 40 mL/min during the measurement. The DSC was calibrated using indium and sapphire. The data were analyzed using the TRIOS v5.1.0.46403 software of TA Instruments.

#### 2.8.2. Powder X-Ray Diffraction (PXRD)

The crystal form of the sample powder was determined using an Ultima IV X-ray diffractometer (Rigaku, Matsubara-cho Akishima-shi, Tokyo, Japan) with Cu Kα radiation (1.541836 Å) at room temperature. The powder was placed on a glass sample holder and pressed with a semicircular stainless-steel plate to achieve a flat surface with a uniform powder distribution. Diffraction patterns were measured using a tube voltage of 40 kV and a current of 40 mA. The divergence slit and anti-scattering slit settings were set at 0.5°. Scans were performed between 3° and 40° in 2θ with steps of 0.02° and a count time of 0.1 s per step.

#### 2.8.3. Scanning Electron Microscopy (SEM)

The appearance and morphology of the samples were studied using a field emission scanning electron microscope (SEM, TM4000PLUS, Hitachi, Tokyo, Japan). Before the experiment, the samples were placed on a substrate and coated with gold particles by sputtering. The acceleration voltage used was 15 kV.

#### 2.8.4. Fourier Transform Infrared (FTIR)

The interactions between the components of the complex were investigated by FTIR spectroscopy. The test was performed at room temperature using a FTIR spectrometer (Thermo Scientific, Waltham, MA, USA). The ATR module was used to collect data and subtract the air background. Subsequently, the sample powder was placed in the circular hole of the ATR. The scan range was set from 500 to 4000 cm^−1^ at a resolution of 2 cm^−1^, and 64 scans were collected.

#### 2.8.5. Dynamic Vapor Sorption (DVS) Analysis

The hygroscopicity of the complex was determined using a Dynamic Vapor Sorption instrument (SMS Ltd., London, UK) at 37 °C. Approximately 30 mg of the powder was weighed into an aluminum pan. The humidity of the incubator was controlled using saturated water vapor and flowing nitrogen gas. The method stage type was set to step dm/dt [%/min] mode. The next step proceeds when the value of dm/dt ≤ 0.002. The sample was initially exposed to 0% relative humidity (RH) and then increased to 90% RH in a step size of 10% increments.

### 2.9. In Vitro Dissolution Study

#### 2.9.1. Non-Sink Dissolution

Non-sink dissolution testing is an effective method for obtaining supersaturated formulations [34]. The non-sink dissolution protocol was based on previous studies [32]. PBS (100 mL, pH 6.8) was added to a 250 mL iodine flask. The flasks were placed in a water bath stirrer (ZNCL-GS, Gongyi Instrument, Gongyi, China) with constant temperature function and preheated at 37 °C for 2 h. After equilibrium, the complex (containing approximately 10 mg of LC001) was added to the medium. The dissolution medium was stirred at 150 rpm. At scheduled time intervals (5, 15, 30, 60, 90, and 120 min), 1 mL of solution was extracted using a sampler with a filter (material: PE, pore size: 0.45 μm), and 1 mL of acetonitrile was added for dilution. The same volume of fresh medium was promptly replenished into the tubes. The sample solution was vortexed (XH-D Vortex Mixer, Tenlin Instrument, Yancheng, China) for 10 s. The concentration of dissolved LC001 was determined using the method described in Section 2.2. All samples were prepared in triplicate.

#### 2.9.2. Dissolution Studies Using the pH-Shift Method

Dissolution testing may be effective for neutral and acidic drugs in a single intestinal medium (such as PBS or FaSSIF media). However, the increased solubility of poorly soluble weak basic drugs at gastric pH should be evaluated during formulation development. Additionally, the variability in gastric pH, which affects drug dissolution and absorption, needs to be considered [20,35,36]. Therefore, the pH-shift dissolution method [37] was employed to simulate the transfer of drugs from a simulated gastric to a simulated intestinal environment. The complex powder (containing approximately 25 mg LC001) was added to 50 mL of pH 1.2 or 4.5 medium. The dissolution medium was preheated to 37 °C for 2 h to reach equilibrium. Within the first 30 min, 1 mL of the solution was extracted at scheduled intervals (5, 10, 20, and 30 min), filtered through a 0.45 µm membrane filter, and diluted with 1 mL of acetonitrile. An equivalent volume of fresh medium was promptly replenished into the flask. After 30 min, 50 mL of a highly concentrated phosphate buffer was added, and the pH of the dissolution medium was immediately raised to 6.8. Samples were collected at 5, 15, 30, 60, 90, 120, and 180 min after the buffer solution was changed, and the concentration of LC001 dissolved in the phosphate buffer was determined. A 1 mL sample was withdrawn, filtered through a 0.45 µm membrane filter, and then diluted with 1 mL of acetonitrile. The absorbance of the solution at 263 nm was measured using a UV-Vis spectrophotometer. The absorbance of the corresponding blank solvent was subtracted, and the concentration of LC001 in the solution was calculated using the external standard method. All experiments were conducted at 37 ± 0.5 °C and repeated three times. After the dissolution process, the pH of the solution was measured to ensure it was 6.8 ± 0.5. To enhance the comparability of the graph, half of the concentration measured in the pH 1.2 medium was displayed in the graph for the period before 30 min. The volume of the dissolution medium was increased from 50 mL to 100 mL. Consequently, even if the drug is completely dissolved in the medium, the concentration would be reduced by half.

### 2.10. Stability Testing

The powder of formulation F3 was stored in a sealed container under long-term conditions at 25 °C/60% relative humidity for 3 months using a constant temperature and humidity chamber (Binder, Germany). Differential scanning calorimetry (DSC, see Section 2.8.1) and power X-ray diffraction (P-XRD, see Section 2.8.2) were employed to analyze the sample state. In vitro dissolution tests (see Section 2.9.2) were performed to evaluate the stability of the amorphous ternary complex formulation.

### 2.11. Statistical Analysis

Data analysis and graphing were performed using Origin Pro 9.0 (Origin Lab Corporation, Northampton, MA, USA). Data are expressed as means and standard deviations.

## 3. Results and Discussion

### 3.1. Physicochemical Properties of LC001

#### 3.1.1. Solubility of LC001 in Different pH Medium

The pH values of the data points in the figure represent the pH of the saturated supernatant containing LC001 after shaking for 24 h. The solubility of LC001 exhibited a pH dependence characteristic of weakly basic drugs. The solubility of LC001 decreased as the pH of the solution increased. The concentration of LC001 was 6.07 ± 0.13 mg/mL in an acidic medium at a pH of 1.39. With an increase in pH value, the solubility decreased to 0.19 ± 0.01 mg/mL in pH 3.0 medium and decreased to 0.016 ± 0.006 mg/mL in the intestinal pH environment (pH = 6.8). Owing to the presence of amino groups in the molecular structure, LC001 exhibits weak basicity, with a pKa value of 3.22. In solutions where the pH is below the pKa, the amino group ionizes, causing LC001 to exist in an ionized form with higher solubility. As the pH increased, the degree of ionization decreased. When the solution pH exceeds the pKa, ionized LC001 is converted into a poorly soluble weak base molecule. The pH-dependent solubility of weakly basic LC001 arises from its varying degrees of ionization in media with different pH levels. Supersaturated drug solutions may be generated when the dissolved drug moves from the stomach to the small intestine. Therefore, precipitation inhibitors are required to maintain the effective concentration of the drug and prevent precipitation.

Additionally, the variability in gastric pH among patients and between fasting and fed states may lead to higher pharmacokinetic variability for drugs with pH-dependent solubility after oral administration [20,35]. Consequently, it is essential to design formulations that enhance the solubility of LC001 in the intestinal environment while avoiding the impact of pH changes in the gastric environment on oral bioavailability.

#### 3.1.2. Thermal Analysis and PXRD of LC001 Powder

**Figure 1:**
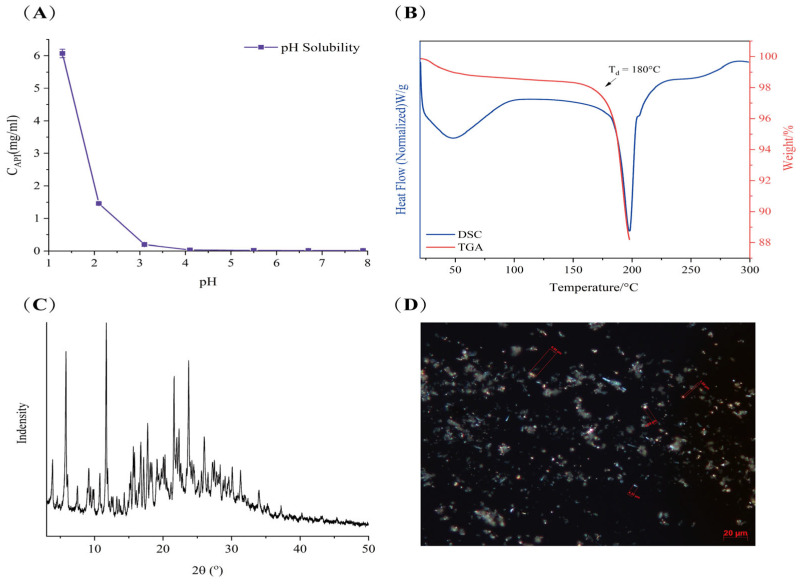
Characterization of LC001: (**A**) pH solubility profile; (**B**) thermogravimetric analysis and differential scanning calorimetry; (**C**) powder X-ray diffraction; and (**D**) polarized light microscope images (500×) of LC001.

The TGA and DSC spectra of LC001 are presented in Figure 1B. The DSC curve exhibited a broad endothermic peak due to the evaporation of moisture in the sample at temperatures below 100 °C. As the temperature increased to 180 °C, a significant inflection point appeared in the TGA signal, indicating a sharp decrease in the weight percentage of the sample caused by decomposition. At the same temperature, the DSC signal exhibited a clear inflection point with an endothermic peak, suggesting that the melting of LC001 was accompanied by decomposition. The PXRD pattern of LC001 displayed distinct diffraction peaks for the crystalline form, as illustrated in Figure 1C. Polarized light microscopy images revealed irregular rod-shaped crystals of the API powder with particle sizes less than 10 μm, as depicted in Figure 1D.

### 3.2. Phase Solubility Study

**Figure 2:**
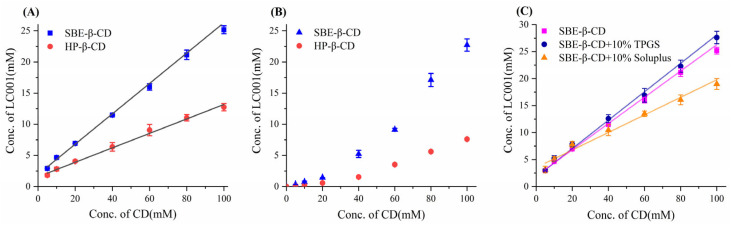
Phase solubility diagram of LC001 in SBE-β-CD and HP-β-CD solutions: (**A**) aqueous medium; (**B**) phosphate buffer medium (pH 6.8), and (**C**) SBE-β-CD aqueous medium in the presence of 10% (*w*/*w*) TPGS and Soluplus. The straight lines in the graph represent the fitted lines of the data points. (Mean ± SD (*n* = 3)).

The phase solubility diagrams of LC001 with cyclodextrin are shown in Figure 2. The complexation with cyclodextrin significantly improved the solubility of LC001. Within the cyclodextrin concentration range of 5–100 mmol/L, the solubility of LC001 increased from 2.9 mmol/L to 21.5 mmol/L and from 1.9 mmol/L to 12.3 mmol/L in the presence of SBE-β-CD and HP-β-CD, respectively. Compared to its solubility in pure water, the solubility of LC001 was enhanced by approximately ~430 times and ~247-fold in 100 mmol/L cyclodextrin solution, respectively. As shown in the phase solubility diagram in an aqueous medium (Figure 2A), the solubility of LC001 increased linearly with the concentration of cyclodextrin (CD) within the studied concentration range. The fitting results of the phase solubility curves are presented in Table S1. The calculated values of *K*_S_, CE, and D: CD based on the fitting results are listed in Table 2. The results of *K*_S_, CE, and D: CD calculated from the fitting results are detailed in Table 1. The *K*_S_ and CE values of SBE-β-CD and LC001 were higher than those of HP-β-CD. The D: CD values indicated that the same solubilizing effect could be achieved using less SBE-β-CD compared to HP-β-CD. The phase solubility curves of LC001 and cyclodextrin in PBS (pH 6.8) are shown in Figure 2B. The saturated solubility of LC001 increased significantly with rising SBE-β-CD concentrations. The phase solubility curves of LC001 with cyclodextrin, which deviate from linearity, can be classified as Ap-type phase solubility diagrams [27]. This may be attributed to the aggregation of cyclodextrin at high concentrations in PBS. The positive curvature of the phase solubility curve indicates the presence of a soluble complex with D:CD < 1 in the solution, where multiple CD molecules form complexes with a single LC001 molecule at higher CD concentrations. Therefore, when the concentration of cyclodextrin exceeds its critical aggregation concentration, it can provide better solubility for poorly soluble drugs. However, the use of high concentrations of cyclodextrin may also lead to permeability issues [38]. When using cyclodextrins for solubilization, it is necessary to consider the balance between concentration and permeability. The solubilizing effect of SBE-β-CD on LC001 was significantly more substantial than that of HP-β-CD in both aqueous medium and PBS, indicating that SBE-β-CD is the optimal choice for enhancing the solubility of LC001.

Owing to the limited precipitation inhibition effect of SBE-β-CD on LC001 (Section 3.5.1), the ternary LC001-SBE-β-CD-polymer complex system was considered. The effects of water-soluble polymers TPGS and Soluplus on the complexation between SBE-β-CD and LC001 were investigated. As shown in Figure 2C, the addition of 10% (*w*/*w*) polymers had different effects on the complexation of LC001 with SBE-β-CD. When TPGS was added, both the *K*_S_ and CE values of the ternary system increased, indicating that TPGS exerted a synergistic complexing effect. The decrease in the D: CD ratio suggested that the introduction of TPGS could reduce the amount of SBE-β-CD. In contrast, Soluplus had the opposite effect, possibly due to its competition with LC001 in complexing with SBE-β-CD. Therefore, the solubilization effects of SBE-β-CD/TPGS solutions with different molar ratios on LC001 were further studied.

### 3.3. Molecular Docking Simulation

**Figure 3:**
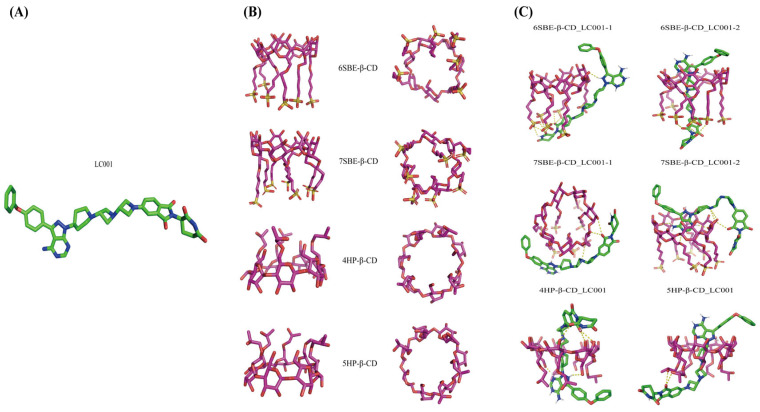
Molecular docking simulation: (**A**) the optimized stable structure of LC001 (red: C-O bonds; blue: C-N bonds; green: C-C bonds); (**B**) different orientations of optimized structures of SBE-β-CD and HP-β-CD with different degrees of substitution; and (**C**) the stable complex conformation of LC001 docked with SBE-β-CD and HP-β-CD molecules. The yellow dashed lines indicate the hydrogen bonds formed between LC001 and CD.

Molecular docking simulation allows us to understand the interactions between ligands and receptors, which is beneficial for comprehending host-guest interactions. The docking study calculated the binding affinity between LC001 and SBE-β-CD and predicted the optimal binding conformation between the host and guest. Figure 3A,B displays the optimized stable structures of LC001, SBE-β-CD, and HP-β-CD with varying degrees of substitution. The docking conformations of LC001 with SBE-β-CD and HP-β-CD at different degrees of substitution are shown in Figure 3C. The binding affinity values in Table 3 indicate stable complex structures, and the negative binding affinity values of all complex conformations suggest that docking reactions were spontaneous [31]. The RMSD values of the docking results were approximately 2.2, demonstrating the reliability of the docking results. Figure 3C shows that the complexation between SBE-β-CD and LC001 involves inclusion and non-inclusion conformations. The lower binding energies of the non-inclusion conformations (6SBE-β-CD_LC001-1 and 7SBE-β-CD_LC001-1) indicate that LC001 is more likely to bind to SBE-β-CD in a non-inclusion mode. The spatial hindrance for LC001 to enter the hydrophobic cavity of SBE-β-CD is significant because of its long molecular chain and large molecular volume. Consequently, a lower absolute binding energy of 1.39 kcal/mol was obtained for the inclusion conformation (6SBE-β-CD_LC001-2). Due to the absence of one sulfobutyl chain on the glycosidic chain of the 6-substituted SBE-β-CD molecule, the LC001 molecule was inserted into the side chain of SBE-β-CD from the site where the sulfobutyl chain was missing, forming a hydrogen bond interaction with the oxygen atom on the sulfonyl group. The LC001 molecule was attached to the sulfobutyl chain of SBE-β-CD because of the steric hindrance of the 7-substituted sulfobutyl group, forming a stable conformation (7SBE-β-CD_LC001-1) with a non-inclusion binding energy of −6.198 kcal/mol. The conformation of the non-inclusion complex formed by the interaction of the drug with the sulfobutyl side chain of SBE-β-CD was also reported by Marta et al. [39]. In addition, because the pyrazole and pyrimidine groups of the LC001 molecule were encapsulated within the hydrophobic cavity of SBE-β-CD, a stable conformation with a binding energy of −5.925 kcal/mol was formed (7SBE-β-CD_LC001-2). Intermolecular hydrogen bonds were identified between the -NH_2_ and -C=O groups in the LC001 molecule and the -OH groups in the SBE-β-CD molecule. Owing to the minor steric hindrance of the hydroxypropyl group, the LC001 molecule can more easily enter the hydrophobic cavity of HP-β-CD. The absolute values of the binding energy between LC001 and HP-β-CD with two different degrees of substitution were higher than 7 kcal/mol. Therefore, steric hindrance may be the primary obstacle preventing the formation of inclusion complexes between LC001 and the CD molecules.

Although molecular docking helps us understand the binding mode between the host and guest, it is a simulation conducted at the single molecule scale. In reality, the binding of LC001 with CD in solution forms complex aggregates with a CD:D ratio greater than one. Molecular dynamics simulation is necessary to further understand the aggregation behavior of larger complexes.

### 3.4. Characterization of the LC001-SBE-β-CD Binary System

#### 3.4.1. DSC, TGA, and PXRD of LC001-SBE-β-CD

**Figure 4:**
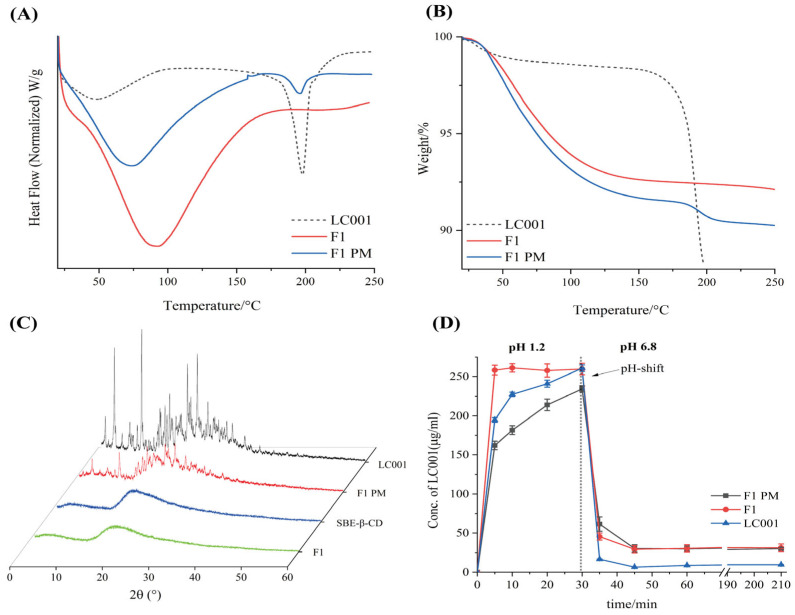
Characterization of LC001-SBE-β-CD binary system: (**A**) differential scanning calorimetry; (**B**) thermogravimetric analysis; (**C**) powder X-ray diffraction patterns of the LC001-SBECD binary system and (**D**) in vitro dissolution profiles of LC001, formulation F1, and F1PM using the pH-shift method, where the pH of the dissolution medium changes from 1.2 to 6.8. The vertical black dashed line in the figure indicates the time of the pH transition. (Mean ± SD, *n* = 3).

As shown in Figure 4A, an endothermic peak corresponding to the melting point of LC001 was observed in the DSC curve of the PM. The broad endothermic peak below 150 °C in the complex was attributed to the volatilization of moisture from the powder, the evaporation of maleic acid in the API, and the dehydration of SBE-Β-CD molecules [33]. No crystalline melting peak of LC001 was detected above 150 °C, indicating the transformation of LC001 into an amorphous form, which was confirmed by the PXRD pattern (Figure 4C). The PXRD pattern of SBE-β-CD did not exhibit distinct, sharp, crystalline characteristic peaks. Although the characteristic peak of the crystallization of LC001 appeared in the binary physical mixture, it was not observed in the complex (F1), confirming the formation of amorphous LC001. As shown in Figure 4B, F1 did not show a significant weight loss inflection at 180 °C compared to the physical mixture, indicating the enhanced thermal stability of LC001 in the complex.

#### 3.4.2. In Vitro Dissolution Study

The pH-transfer dissolution method was used to simulate and study the transport of weakly alkaline drugs from the low pH gastric environment to the high pH intestinal environment, which can more accurately reflect the impact of gastric pH changes on dissolution performance [35,40,41]. The pH-transfer dissolution profiles of LC001, F1 PM, and F1 are shown in Figure 4D. LC001 powder and physical mixture exhibited relatively slow dissolution rates in a pH 1.2 medium. The dissolution concentration of LC001 powder (half value of the actual dissolved concentration) increased from 194.5 ± 3.1 µg/mL to 260.8 ± 4.1 µg/mL within 5–30 min. F1 showed the fastest dissolution rate, reaching the dissolution plateau in 5 min, where the measurement of LC001 concentration was 258.3 ± 4.1 µg/mL. The physical mixture exhibited the slowest dissolution rate and was not completely dissolved, with an LC001 concentration of 234.3 ± 4.0 µg/mL detected at 30 min. The undissolved solid particles were observed in the physical mixture, likely due to the aggregation of SBE-β-CD forming a colloidal solid, insoluble substance in the presence of API, which hindered the dissolution of API. When a high-concentration phosphate buffer was added to the pH 1.2 medium, the solution pH suddenly increased to 6.8, leading to a sharp decline in the concentration of API dissolution. A low API concentration of 6.5 ± 0.1 µg/mL was detected. Due to the weak precipitation inhibition effect of SBE-β-CD, slightly higher API concentrations of approximately 30 µg/mL in F1 PM and F1 were detected. Compared to the physical mixtures, the complex did not exhibit a dissolution advantage, resulting in a lower effective concentration of LC001 in the intestine, which is unfavorable for improving bioavailability.

Owing to the poor precipitation-inhibiting effect of the SBE-β-CD solution on LC001, the binary LC001-SBE-β-CD complex failed to enhance the solubility and dissolution rate of the API in PBS (pH 6.8). Consequently, the next step was to incorporate a precipitation inhibitor to create an LC001-SBE-β-CD-polymer ternary complex, with the aim of enhancing the dissolution rate and improving the solubility of LC001 in the intestine.

### 3.5. Screening of Precipitation Inhibitors

#### 3.5.1. Saturation Solubility and Precipitation Inhibition Studies of LC001 in Different Hydrophilic Polymers

**Figure 5:**
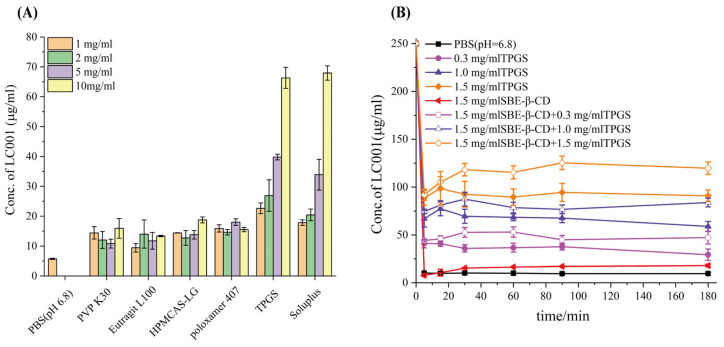
Screening of precipitation inhibitors: (**A**) solubility of LC001 in polymers pre-dissolved in PBS (pH 6.8) at different concentrations; and (**B**) precipitation rate of LC001 in PBS (pH 6.8) containing various concentrations of TPGS and mixtures of SBE-β-CD and TPGS. (Mean ± SD, *n* = 3).

A supersaturated drug solution can form when dissolved drugs pass through the stomach and enter the small intestine. A stable and effective supersaturated concentration is necessary for intestinal absorption of drugs. Maintaining a high drug concentration in the intestinal lumen is essential for improving bioavailability [35,42]. Consequently, polymer precipitation inhibitors commonly employed in ASD systems were screened to improve the solubility of LC001 in PBS (pH 6.8) in the presence of SBE-β-CD. First, the saturated solubility of LC001 in different polymers prepared in pH 6.8 medium at varying concentrations was studied. As shown in Figure 5A, the solubility of the API in PBS (pH 6.8) was 5.7 ± 0.2 µg/mL. The water-soluble polymers PVP K30, Eudragit L100, HPMCAS-LG, and Poloxamer 407 did not significantly improve the solubility of LC001, with only a low concentration of approximately 15 µg/mL of LC001 detected. Different concentrations of TPGS and Soluplus significantly enhanced the solubility of LC001. The highest concentration of LC001 (22.6 ± 1.8 µg/mL) was detected in a 1 mg/mL TPGS solution. Precipitation inhibition of LC001 by SBE-β-CD solutions at varying concentrations and mixed solutions of SBE-β-CD and TPGS was investigated. In the mixed solutions, the concentrations of SBE-β-CD and TPGS corresponded to the composite formulations F2, F3, and F4 when dissolved in 100 mL of dissolution medium. The solubility of LC001 increased with the rising concentrations of TPGS and SBE-β-CD in the solution (Figure S2). However, as shown in Figure 5B, the 1.5 mg/mL SBE-β-CD solution did not inhibit the precipitation of LC001. When SBE-β-CD was added to TPGS solutions of variable concentrations, the concentration of LC001 increased compared to that in TPGS solutions without SBE-β-CD. As shown in Table S2, the formation of 230–350 nm aggregates in the solutions of TPGS (different concentrations) inhibited the precipitation of LC001. While the aggregates with particle sizes larger than 1 µm were formed in LC001 saturated SBE-β-CD solution. When different concentrations of TPGS were added to the SBE-β-CD solution, stable nano-sized aggregates with lower PDI were formed in the solution.

#### 3.5.2. DSC and In Vitro Dissolution Study of Ternary Complexes with Different TPGS and Soluplus Contents

To investigate the effect of polymer content on the dissolution of the ternary complex, 5%, 10%, and 20% (*w*/*w*) of TPGS and Soluplus were added to the LC001-SBE-β-CD binary complex system. As shown in Figure S3A,B, the DSC signals of the lyophilized powders of the ternary complexes with different polymer contents did not exhibit an endothermic peak at the melting point of the LC001 crystal form, indicating the formation of an amorphous LC001. The dissolution (Figure S3C) of the ternary complexes containing TPGS showed significant differences compared to those containing Soluplus. As the TPGS content increased, the dissolution rate and extent of the complexes improved significantly. The dissolution of the ternary complex with 20% (*w*/*w*) TPGS reached approximately 90% within 10 min. However, the dissolution was below 20% for complexes with different Soluplus contents. The particle sizes of the complex solutions with different polymer contents are presented in Table S2. Nanoparticles with low PDI values and a particle size of 152–177 nm were detected in the dissolution media of ternary complexes with varying TPGS content. Aggregates with higher PDI values and particle sizes in the micrometer range were detected in the dissolution of the complex containing Soluplus, indicating poor dispersion of nanoparticles and weak stability of the LC001 aggregates formed in the dissolution medium. Therefore, the dissolution performance of the complex containing Soluplus was inferior to that of TPGS. Consequently, the next step of the research was optimizing the SBE-β-CD/TPGS ratio in the formulation.

### 3.6. Screening of the Molar Ratio of SBE-β-CD/TPGS for the Preparation of Ternary Complexes

**Figure 6:**
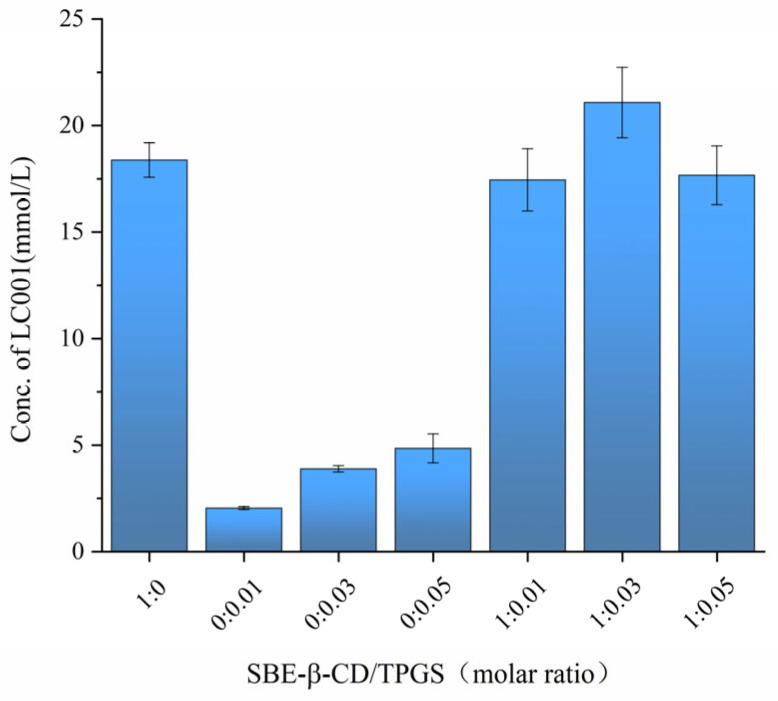
Saturated solubility of LC001 in mixed solutions of SBE-β-CD and TPGS at different molar ratios. (Mean ± SD, *n* = 3).

To investigate the impact of different SBE-β-CD/TPGS ratios on the solubilization effect of LC001, the saturated solubility of LC001 in solutions with varying molar ratios of SBE-β-CD/TPGS was measured. As shown in Figure 6, the saturated solubility of LC001 in a 50 mmol/L SBE-β-CD solution was 18.05 ± 1.25 mmol/L, while its solubility in TPGS solutions without SBE-β-CD was consistently less than 5 mmol/L. The highest solubility of LC001, reaching 21.08 ± 1.65 mmol/L, was observed in a mixed solution with an SBE-β-CD/TPGS molar ratio of 1:0.03. Consequently, this molar ratio was selected to prepare the ternary inclusion complex, which facilitated the reduction in the amount of SBE-β-CD used in the formulation.

The particle sizes of the binary LC001/SBE-β-CD and ternary LC001/SBE-β-CD/TPGS complexes were evaluated using DLS. The particle size and zeta potential of the saturated solutions are summarized in Table S3. Three particle size distributions were detected in the binary LC001-SBE-β-CD solution. These were 0.83 ± 0.10 nm with an intensity of 19.57 ± 0.59, the formed LC001/SBE-β-CD inclusion complex of 2.29 ± 0.19 nm with an intensity of 61.7 ± 6.11, and large inclusion aggregates of 235.67 ± 78.30 nm with an intensity of 18.77 ± 4.80. The diameters of the CD aggregates mainly ranged between 90 and 300 nm, but the size distribution spanned from 20 nm to several micrometers [43]. These results are consistent with those of previous studies [17,33].

The particle sizes of the ternary complexes (LC001-SBE-β-CD-TPGS) with different SBE-β-CD/TPGS molar ratios were larger than those of the binary systems. The sizes of the ternary complexes exhibited multiple distributions, consisting of complex aggregates with particle sizes of 10.67~18.22 nm. Micellar aggregates with particle sizes of 343.07~736.40 nm may also form due to the presence of TPGS in the system [44]. The micellar aggregates formed in the solution with an SBE-β-CD/TPGS molar ratio of 1:0.03, exhibiting a maximum intensity of 78.33 ± 3.52%, may be the reason for the improved solubility. In addition, the TPGS structure contains a large number of polyoxyethylene units that can stabilize the LC001/SBE-β-CD complex through steric effects [33]. It is possible that the higher the absolute value of the zeta potential in a nanoparticle system, the greater the stability of that system. The zeta potential values of the nano aggregates in the LC001/SBE-β-CD/TPGS ternary complexes were lower than those in the binary complexes; however, their stability was maintained by van der Waals forces, hydrogen bonding interactions, and the steric hindrance effects of the polymers [43].

### 3.7. FTIR and DVS Study

**Figure 7:**
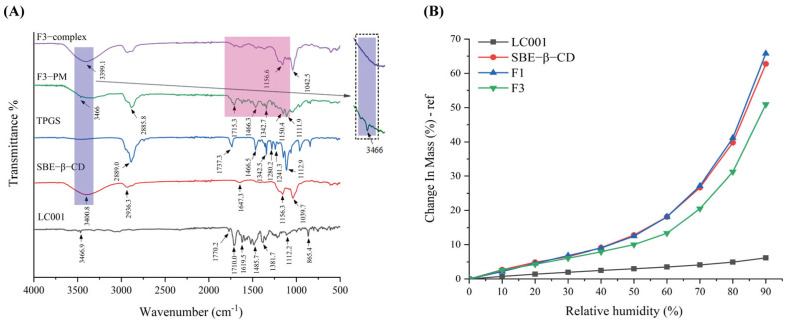
Interaction studies: (**A**) infrared spectra of pure LC001, SBE-β-CD, TPGS, formulation F3, and the physical mixture (F3 PM); (**B**) DVS curves of pure LC001, SBE-β-CD, and complex formulations F1 and F3.

The interactions between the components of the complex were evaluated by FTIR and the results are presented in Figure 7A. LC001 exhibited characteristic peaks at 3467 cm^−1^, 1770 cm^−1^, and 1710 cm^−1^, corresponding to the N-H stretching vibration of the pyrimidine group and the C=O stretching vibration in the CRBN (thalidomide) group in LC001. The absorption peaks near 1619.5 cm^−1^ and 1485.7 cm^−1^ were attributed to the C=C stretching vibrations of the benzene ring, while the peak at 1381.7 cm^−1^ was ascribed to the C-N stretching vibration in the linker structure of LC001. The peak at 1112.2 cm^−1^ was associated with the Ar-O stretching vibration. The characteristic stretching peaks of SBE-β-CD were 3400.8 cm^−1^ (O-H stretch), 2936.3 cm^−1^ (C-H stretch), and 1647.3 cm^−1^ (S=O stretch) [45]. Strong absorption peaks were observed at 1156.3 cm^−1^ and 1039.7 cm^−1^, corresponding to C-H and C-O stretching vibrations, respectively [46]. The TPGS spectrum exhibited a strong methylene stretching vibration peak at 2889.0 cm^−1^, a C=O stretching vibration at 1737.3 cm^−1^, a strong peak at 1466.5 cm^−1^ ascribed to -CH_2_ bending vibration, and an absorption band at 1112.9 cm^−1^ attributed to C-O-C stretching vibration in the polyoxyethylene unit [44,47,48]. The infrared spectrum of the binary mixture (F1-PM) was a superposition of the characteristic peaks of SBE-β-CD and LC001, as illustrated in Figure S5. In the infrared spectrum of the F1-complex, the -NH_2_ vibration peak at 3466.9 cm^−1^ in the LC001 molecule disappeared, and the absorption peak at 3387.0 cm^−1^ in the physical mixture shifted to 3392.7 cm^−1^ in the complex, indicating the formation of intermolecular hydrogen bonds between the SBE-β-CD molecule and -NH_2_ groups in LC001. The weakening or disappearance of the absorption band at 1709.0–1485.2 cm^−1^ in the infrared spectrum of the F1-complex suggested that the formation of the inclusion complex restricts the molecular vibrations of LC001.

Due to the low energy of molecular motion in the physical mixing preparation method, which is insufficient to induce intermolecular collisions [49], the superposition of the characteristic vibration peaks of TPGS, SBE-β-CD, and LC001 was observed in the spectrum of the ternary physical mixture (F3 PM), as shown in Figure 7A. In the complex spectrum, the -NH_2_ vibration peak of LC001 at 3466.9 cm^−1^ disappeared, and the O-H vibration peak of SBE-β-CD at 3400.8 cm^−1^ shifted to 3399.1 cm^−1^, indicating the presence of intermolecular hydrogen bonding interactions in the inclusion complex. The disappearance of the absorption band at 1715.3 cm^−1^–1342.7 cm^−1^ in the complex suggests the formation of an inclusion complex. The disappearance of the stretching vibration peak of TPGS at 1112.9 cm^−1^ in the spectrum of F3-complex indicates that the oxyethylene group of TPGS acted as a hydrogen bond acceptor, forming intermolecular hydrogen bonds with LC001 or SBE-β-CD molecules. The formation of hydrogen bonds may be the reason why TPGS contributes to the stabilization of the binary complex.

Figure 7B displays the DVS curves of LC001, SBE-β-CD, and inclusion complex (F1 and F3) powders. LC001 exhibited weak hygroscopicity, with a weight change rate of less than 10% at 90% relative humidity (RH). The weight change in the SBE-β-CD powder increased exponentially as the relative humidity increased. The significant hygroscopicity observed in the binary complex was primarily due to the highly hygroscopic characteristics of SBE-β-CD. The introduction of TPGS in the formulation reduced the weight change rate of the ternary complex. This reduction is likely due to the formation of intermolecular hydrogen bonds that occupy the solvation sites of SBE-β-CD [50]. Given the strong hygroscopicity of SBE-β-CD, it is essential to store the solid composite powder in a low-humidity environment or implement proper drying measures to prevent the deliquescence of the sample.

### 3.8. Surface Morphology

**Figure 8:**
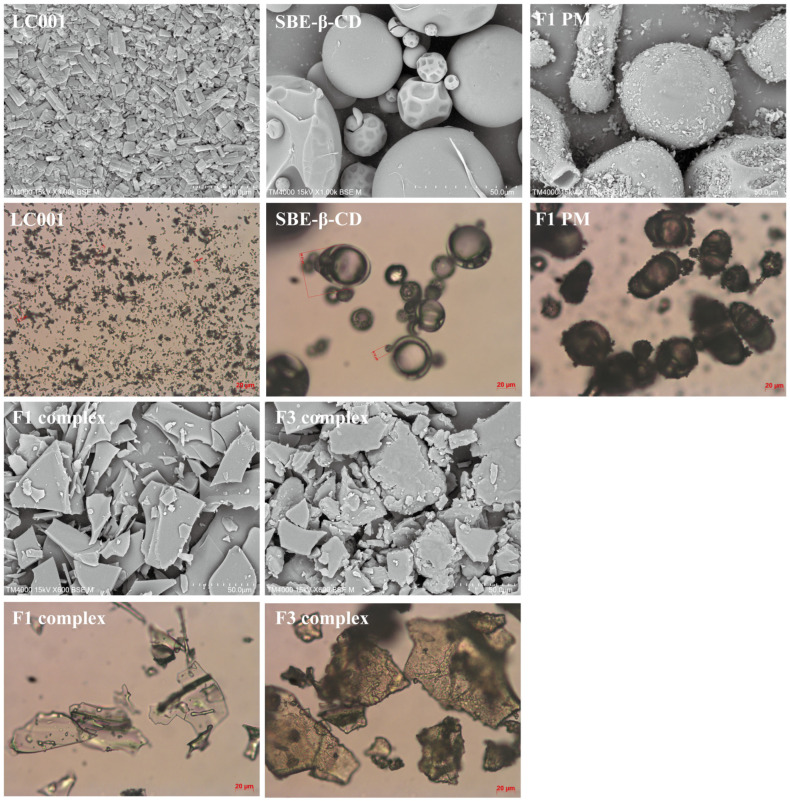
Scanning electron microscopy images and micrographs of pure LC001, SBE-β-CD, binary physical mixture (F1-PM), and binary and ternary complex formulations F1 and F3.

Figure 8 shows the surface morphology of LC001, SBE-β-CD, the binary physical mixture of LC001-SBE-β-CD (F1-PM), the binary complex (F1 complex), and the ternary complex of LC001-TPGS-SBE-β-CD (F3 complex). LC001 exhibited irregular rectangular or block-shaped crystals. The irregular spherical particles with a hollow interior and wrinkled exterior in the size range of 10–50 µm were observed in the SEM images of SBE-β-CD. The SEM image of F1PM reveals the morphology of the mixture formed by LC001 adsorbed onto the spherical surface of SBE-β-CD. Unlike the smooth and flat appearance of the binary complex, a rough surface was formed in the ternary complex in the presence of TPGS. In addition, the transparency of the ternary complex powder was reduced, as observed under a microscope. The disappearance of the specific morphological characteristics of SBE-β-CD and LC001 and the formation of irregular amorphous solids indicated the formation of the complex.

### 3.9. Characterization of Ternary Complexes

#### 3.9.1. DSC and PXRD

**Figure 9:**
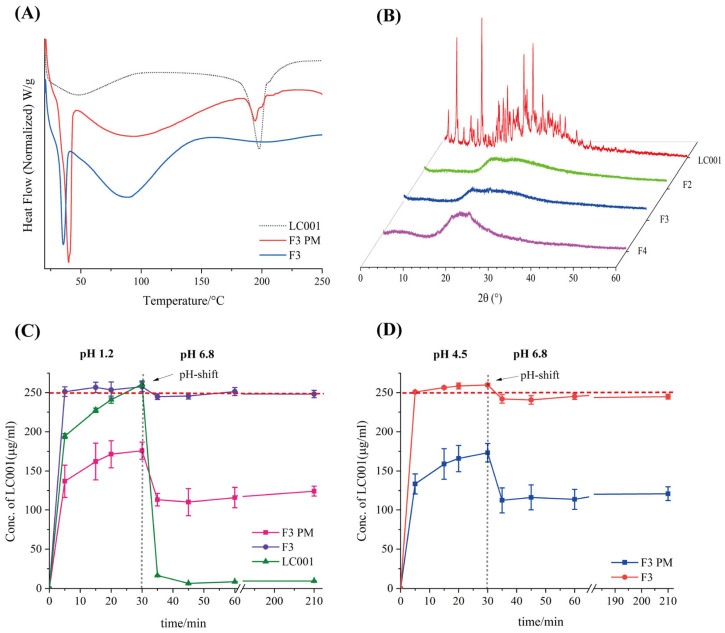
Characterization of LC001-SBE-β-CD ternary system by (**A**) differential scanning calorimetry thermograms of formulation F3 and the physical mixture of F3 (F3 PM); (**B**) powder X-ray diffraction patterns of complex formulations F2, F3, and F4; (**C**,**D**) pH-shift dissolution profiles of F3 and F3 PM; (**C**) the pH of the dissolution medium changes from 1.2 to 6.8; (**D**) the pH of the dissolution medium changes from 4.5 to 6.8. The vertical dashed lines in the figures indicate the time points of the pH shift. The horizontal red dashed lines correspond to the concentrations at which LC001 was completely dissolved. (Mean ± SD, *n* = 3).

As shown in Figure 9A and Figure S4A, the strong endothermic peaks observed below 50 °C for both the complex and the physical mixture can be attributed to the melting of TPGS. The DSC signal of the physical mixture exhibited an inflection point at the melting point of LC001. In contrast, the signal of the complex did not significant change, indicating the formation of amorphous LC001, which was also confirmed by the XRD results (Figure 9B). The absence of distinct characteristic peaks of LC001 in the XRD patterns, with only halo patterns observed, indicated the formation of amorphous complexes in F2, F3, and F4.

#### 3.9.2. In Vitro Dissolution

Figure S4B illustrates the dissolution profile of the ternary complex in PBS (pH 6.8). Due to the weak precipitation inhibition effect of SBE-β-CD, the binary complex F1 reached a dissolution plateau after 15 min, with only about 10% of LC001 dissolved. The dissolution in the presence of TPGS alone at different concentrations (F2 (without CD) and F3 (without CD)) was below 20%. Consistent with the precipitation inhibition results discussed in Section 3.5.1, which TPGS or SBECD alone exhibited weak precipitation inhibition effects on LC001. In contrast, ternary complexes with varying TPGS contents showed a notable improvement in dissolution, with at least 80% of LC001 dissolving within 5 min and reaching a plateau by 30 min. The approximately 100% dissolution of LC001 in the ternary complexes (F2 and F3) indicates that preparing the ternary complex is necessary to improve the dissolution behavior of LC001.

Considering pH-dependent solubility, LC001 may initially dissolve in the acidic conditions of the stomach and then rapidly precipitate upon entering the small intestine, potentially compromising bioavailability and leading to pharmacokinetic variability [20,35]. Since the extent of drug precipitation in the small intestine is influenced by supersaturation, gastric conditions such as varying environmental pH levels are particularly critical for oral drug delivery [51]. Therefore, simulating the dissolution and precipitation processes within the human gastrointestinal tract is essential for predicting oral drug absorption and accurately understanding the variability in human drug absorption [40]. The pH-shift dissolution method was employed to investigate the effects of different gastric conditions on the dissolution performance of the complex and its physical mixture (PM) in the intestinal tract. The dissolution profiles of ternary complex F3, its physical mixture, and pure LC001 are shown in Figure 9C,D. Figure 9C illustrates the changes in the dissolution profiles as the pH of the dissolution medium shifted from 1.2 to 6.8. In the medium of pH 1.2, the pure API completely dissolved within 5–30 min, with the detected concentration increasing from 194.5 ± 3.1 µg/mL to 260.8 ± 4.1 µg/mL. When the pH value of the solution was raised to 6.8, LC001 rapidly precipitated, and only 6.5 ± 0.1 µg/mL of LC001 was detected after 45 min. In contrast, the dissolution rate of F3 PM was slower compared to the pure API, with only 175.7 ± 11.1 µg/mL of LC001 detected at 30 min. Similarly to the binary complex formulation F1, large undissolved solid particles were observed in the solution. The slow dissolution rates of physical mixtures may be related to the mixed structure observed in SEM images, where LC001 was adsorbed on the surface of SBE-β-CD spheres. The mixture of SBE-β-CD and TPGS in the dissolution medium did not effectively disperse LC001, leading to the aggregation and crystallization of amorphous LC001 (Figure S6A). When the pH value increased to 6.8, the dissolution concentration of F3 PM decreased to 110.1 ± 17.4 µg/mL, similar to the concentration measured in the precipitation inhibition experiment. As the dissolution time increased, the cumulative dissolution concentration increased slightly, with no further precipitation. Compared to the physical mixture, the dissolution rate of the complex was significantly improved. The fully dissolved LC001 concentration of 251.2 ± 6.2 µg/mL (half the actual dissolution concentration) was detected within 5 min. When the pH value increased to 6.8, no significant precipitation of LC001 was observed, and the concentration of LC001 remained ~250 µg/mL without any precipitation throughout the experiment. The same dissolution behavior was observed when the pH of the medium increased from 4.5 to 6.8. The physical mixture dissolved slowly in the medium of pH 4.5. LC001 was not fully dissolved even after 30 min. When a high concentration of phosphate buffer was added, LC001 precipitated and ultimately maintained a concentration of LC001 of 116.1 ± 6.2 µg/mL. The crystal form of LC001 was observed in the dissolution medium (Figure S6B).

In contrast, the complex dissolved rapidly, and no precipitation was observed after the pH transition. The cumulative dissolution concentration detected was approximately twice that of the physical mixture. The enhanced dissolution of the LC001 complex can be attributed to the formation of inclusion complexes, which was confirmed by DSC, FT-IR, and PXRD. However, the improvement in dissolution rate may be attributed to the transition of LC001 from a crystalline form to an amorphous state [33]. In the presence of TPGS, the surface tension between the poorly soluble LC001 and the dissolution medium was reduced, and the formation of stable nanoscale aggregates increased the solubility of the complex. This result is similar to the previously reported solubilization enhancement effect of poorly soluble drugs with ternary complexes formed with hydrophilic polymers and HP-β-CD or SBE-β-CD [52,53,54,55].

Research indicates that size is a critical parameter for controlling the penetration of nanoparticles into gastrointestinal epithelial cells. The uptake of micelles in the intestines and the extent of drug absorption increase as the particle size decreases [36,56]. In this study, binary complex F1 formed precipitates in pH 6.8 medium and particles with a size distribution of approximately 3 μm were detected. Ternary physical mixtures formed aggregates of approximately 190 nm in solution. In contrast, smaller aggregates with a particle size of approximately 78 nm were detected in the dissolution medium of ternary complex F3 (Figure S7), possibly due to the formation of micelle-like aggregates of TPGS by interacting with the outer surface of SBE-β-CD or LC001/SBE-β-CD complexes, which corresponds to the requirements for absorption in gastrointestinal tissues, leading to better drug absorption [57].

### 3.10. Stability Studies

**Figure 10:**
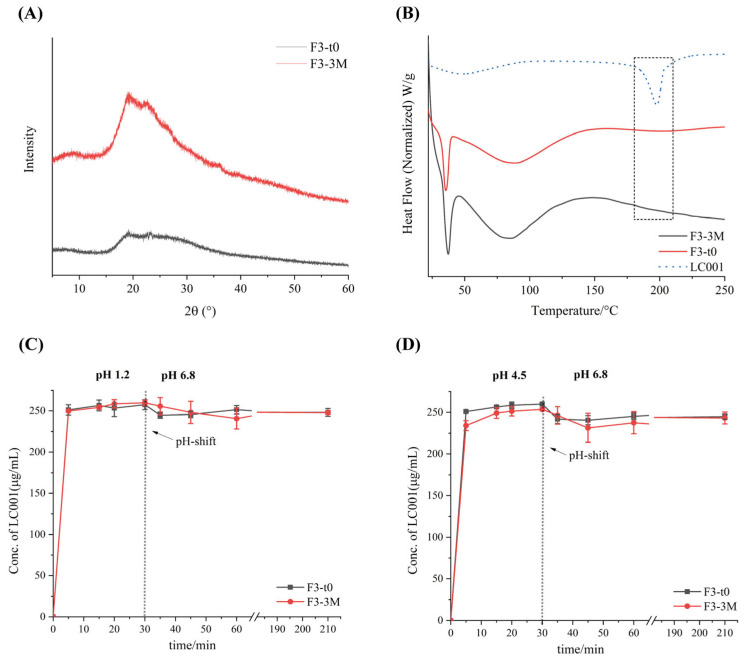
Stability data of formulation F3 at initial time (F3-t0, black line) and after storage for 3 months (F3-3M, red line) under long-term conditions (25 °C/75% RH): (**A**) powder X-ray diffraction (PXRD); (**B**) differential scanning calorimetry (DSC) and two-step dissolution profiles with pH transition; (**C**) pH transition from 1.2 to 6.8 and (**D**) from 4.5 to 6.8. (Mean ± SD, *n* = 3).

The stability of formulation F3 under long-term storage conditions (25 °C/60% RH) in a sealed environment for three months was investigated. As shown in Figure 10, compared to the initial time point (F3-t0), no distinct characteristic diffraction peaks appeared in the P-XRD patterns of the powder after three months of storage (F3-3M), indicating that the API in the powder remained in an amorphous state. This was further confirmed by the DSC thermograms (Figure 10B), where no melting peak corresponding to the crystalline API was observed for either F3-t0 or F3-3M. Compared with the dissolution profile of F3-t0, no significant change was observed in the dissolution profile of F3-3M. Under various pH conditions, formulation F3 maintained rapid dissolution, and no API precipitation occurred after pH transition. All results suggest that the ternary amorphous solid dispersion formulation remained stable after three months of storage under long-term conditions. However, since the current storage duration is relatively short, the long-term storage stability under conditions of 25 °C/60% RH requires further investigation.

## 4. Conclusions

In the present study, Ternary PROTAC-CD complexes were prepared to enhance the solubility and dissolution of LC001, a weakly basic PROTAC drug. SBE-β-CD was screened for its inclusion advantage over LC001 by the phase solubility method. The freeze-drying method was employed to prepare the complexes. The binary LC001-SBE-β-CD complex exhibited poor in vitro dissolution properties, which was associated with the weak precipitation inhibition capability of SBE-β-CD on LC001. Vitamin E-TPGS exhibited significant effects in inhibiting the precipitation of LC001 and enhancing the complexation of SBE-β-CD with LC001. The improved solubility of LC001 in the complex solution was attributed to the formation of micellar aggregates. In addition, the formation of hydrogen bonds between the polyoxyethylene units in the hydrophilic portion of the TPGS and SBE-β-CD stabilized the complex structure. The lack of crystal structure in the lyophilized particles and the formation of nano aggregates in solution may be the reasons for the improved dissolution of the ternary complex.

As far as we know, this study is the first to use cyclodextrin complexation to improve the dissolution performance of a pH-dependent, poorly soluble PROTAC drug. In this sense, promising oral ternary complex formulations has been developed. Further in vivo pharmacokinetic studies are required to validate the improved bioavailability of LC001 in ternary complex formulations.

## Figures and Tables

**Table 1:** Composition of binary and ternary formulations prepared by spray drying.

Formulation	LC001: SBE-β-CD: TPGS (Mass Ratio)	LC001: SBE-β-CD: TPGS (Molar Ratio)
F1	1:9.78:0	1:4:0
F2	1:6.36:1.31	1:3:0.03
F3	1:5.41:3.34	1:2.5:0.07
F4	1:6.18:6.36	1:3:0.14

**Table 2:** Calculated values of complexation constants (*K*_S_) and complexation efficiency (CE) in the binary LC001-SBE-β-CD, LC001-HP-β-CD, and ternary LC001-SBE-β-CD-polymer systems.

	*K*_S_ (mmol/L^−1^)	CE	D:CD
LC001: SBE-β-CD	6.43	0.32	1:4.1
LC001: HP-β-CD	2.64	0.13	1:8.6
LC001: SBE-β-CD: TPGS	7.15	0.36	1:3.8
LC001: SBE-β-CD: Soluplus	3.89	0.19	1:6.1

**Table 3:** Binding affinities and root mean square deviation between LC001 and CDs.

CDs:LC001	Affinity (kcal/mol)	RMSD *
6SBE-β-CD_LC001-1	−6.332	2.347
6SBE-β-CD_LC001-2	−1.39	2.392
7SBE-β-CD_LC001-1	−6.198	2.299
7SBE-β-CD_LC001-2	−5.925	2.298
4HP-β-CD_LC001	−8.121	2.655
5HP-β-CD_LC001	−7.328	2.147

* RMSD: root mean square deviation (calculated by Pymol 2.6.0a0).

## Data Availability

The data is not publicly available at this time as it will be used in other ongoing studies.

## Supplementary Materials

The following supporting information can be downloaded at: https://www.mdpi.com/article/10.3390/pharmaceutics17050671/s1, Table S1: Fitting results of the phase solubility curve; Table S2: The particle size distribution of the dissolution solution of ternary CD complexes containing 5–20% (*w*/*w*) TPGS and Soluplus; Table S3: Particle size and zeta potential of LC001 saturated solutions with different SBE-β-CD/TPGS molar ratios; Figure S1: Chemical structure similar to LC001; Figure S2: Precipitation inhibition of LC001 by different concentrations of (A) TPGS and (B)SBE-β-CD pre-dissolved in PBS (pH 6.8); Figure S3: Differential scanning calorimetry (DSC) thermograms of ternary complexes containing 5–20% (*w*/*w*) of (A) TPGS (B) Soluplus, and (C) dissolution profiles in phosphate buffer medium at pH 6.8; Figure S4: Differential scanning calorimetry (DSC) spectra of pure LC001 and ternary complex formulations F2 and F3 with varying TPGS content; (B) dissolution profiles of binary complex formulation F1, ternary complex formulations (F2, F3) and formulations F2 and F3 without SBE-β-CD in pH 6.8 phosphate buffer medium; Figure S5: Infrared spectra of pure LC001, SBE-β-CD, and LC001-SBE-β-CD binary systems (F1-PM, F1-complex); Figure S6: Undissolved solids of ternary complex (F3) and physical mixture (F3 PM) in the dissolution medium: (A) The pH of the dissolution medium changes from 1.2 to 6.8 and (B) from 4.5 to 6.8.
