# Supplementary material for: Preparation and Characterization of Ternary Complexes to Improve the Solubility and Dissolution Performance of a Proteolysis-Targeting Chimera Drug
Source: Pharmaceutics. 2025 May 20;17(5):671. doi: 10.3390/pharmaceutics17050671 (PMC12115006; doi:10.3390/pharmaceutics17050671)
Supplement: Supplementary file 1 [file pharmaceutics-17-00671-s001.zip › pharmaceutics-3591857-supplementary.pdf]

pharmaceutics

Article

Preparation and Characterization of Ternary Complexes to Improve the Solubility and Dissolution Performance of a Proteolysis-Targeting Chimera Drug

Heng Zhang [1,2,†], Hengqian Wu [2,†], Lili Wang [2], , Laura Machín Galarza [3], Chuanyu Wu [4], Mingzhong Li [5], Zhengping Wang [2,6], , Erpeng Zhou [7], Jun Han [1,2,6]*

[1] School of Chemistry and Chemical Engineering, University of Jinan, Jinan, 250022, China
[2] Institute of Biopharmaceutical Research, Liaocheng University, Liaocheng, 252000, China
[3] Institute of Pharmacy and Foods, University of Havana, 17100, Havana, Cuba
[4] School of Chemistry and Chemical Engineering, University of Surrey, Guildford, GU2 7XH, UK
[5] School of Pharmacy, De Montfort University, Leicester LE1 9BH, U.K.
[6] Liaocheng High-Tech Biotechnology Co., Ltd., Liaocheng, 252000, China
[7] College of Chemical Engineering, Shijiazhuang University, Hebei International Joint Research Center for Biopharmaceutical, Shijiazhuang, 050035, China
* Correspondence: junhanmail@163.com; Tel.: +86-0635-8239136
† These authors contributed equally to this work.

Table S1. Fitting results of the phase solubility curve.

Cyclodextrin	Equation	R2
SBE-β-CD	Y=0.2432x + 1.935	0.9931
HP-β-CD	Y=0.1164x + 1.567	0.9929
SBE-β-CD: TPGS	Y=0.2633x + 1.748	0.9930
SBE-β-CD: soluplus	Y=0.1627x + 3.501	0.9748

Table S2. Particle size of the dissolution solution of ternary CD complexes containing 5%-20% (w/w) TPGS and Soluplus.

Polymer	Mean particle Size/nm	PDI
5% TPGS	176.7±2.6	0.23±0.01
10% TPGS	152.0±1.8	0.11±0.03
20% TPGS	158.9±1.3	0.07±0.01

Academic Editor: Firstname Lastname

Received: date
Revised: date
Accepted: date
Published: date

Citation: To be added by editorial staff during production.

Copyright: © 2025 by the authors. Submitted for possible open access publication under the terms and conditions of the Creative Commons Attribution (CC BY) license (https://creativecommons.org/licenses/by/4.0/).

5% soluplus	1615±147	0.76±0.05
10% soluplus	2169±63.9	0.77±0.06
20% soluplus	792.0±25.8	0.50±0.04

Table S3. The particle size distribution and zeta potential of LC001 saturated solutions with different SBE-β-CD/TPGS molar ratios.

SBE-β-CD/TPGS (molar ratio, C SBE-β-CD=50mM)	Mean particle size (nm; Mean±S.D.)	Intensity (%)	Zeta potential (mV)
1:0	0.83±0.10	19.57±9.91	-5.47±0.59
	2.29±0.19	61.7±6.11	
	61.7±6.11	18.77±4.80	
1:0.01	235.67±78.30	23.87±0.25	-4.96±0.80
	18.22±0.36	76.13±0.25	
1:0.03	636.83±56.62	21.66±3.52	-3.29±0.28
	14.77±1.21	78.33±3.52	
1:0.05	736.40±61.40	28.30±2.23	-3.97±0.62
	10.67±0.59	71.70±2.23	

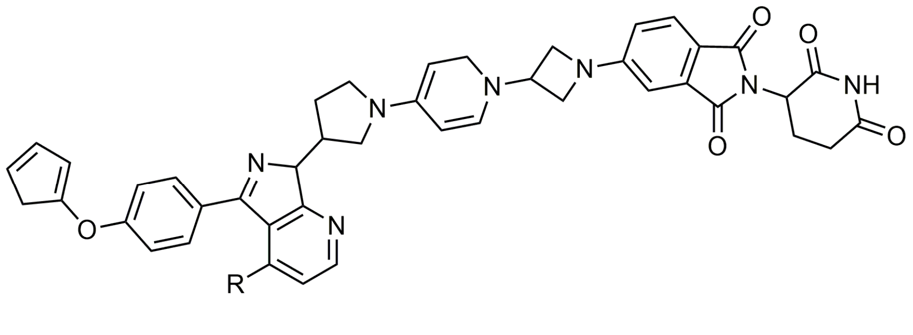

Figure S1. Chemical structure similar to LC001.

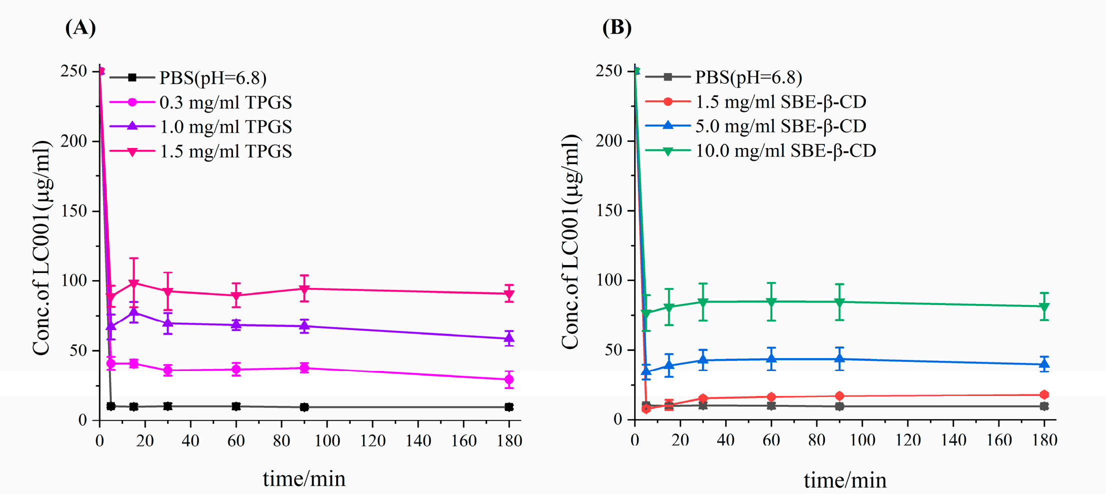

Figure S2. Precipitation inhibition of LC001 by different concentrations of (A) TPGS and (B)SBE-β-CD pre-dissolved in PBS (pH 6.8).

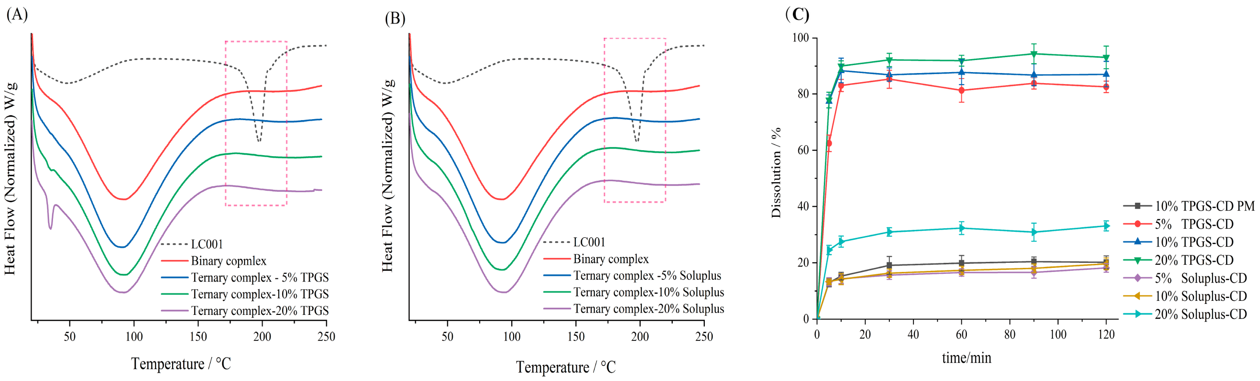

Figure S3. Differential scanning calorimetry (DSC) thermograms of ternary complexes containing 5%-20% (w/w) of (A) TPGS (B) Soluplus, and (C) dissolution profiles in phosphate buffer medium at pH 6.8.

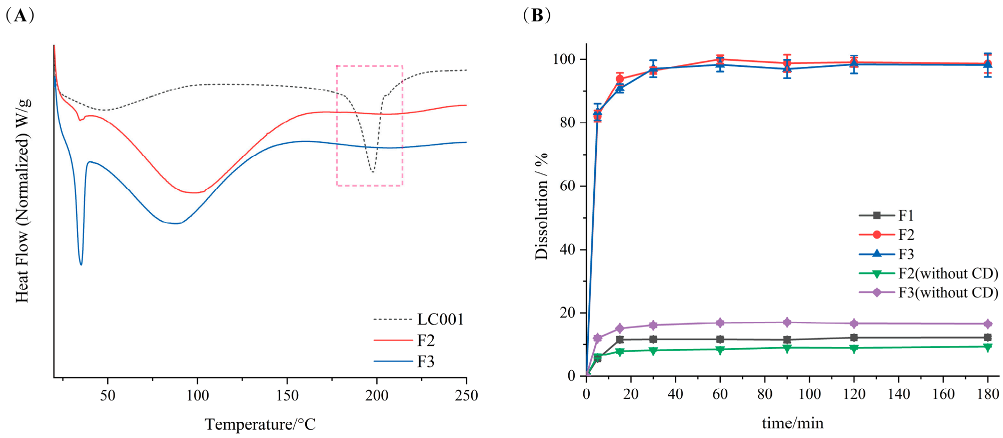

Figure S4. Differential scanning calorimetry (DSC) spectra of pure LC001 and ternary complex formulations F2 and F3 with varying TPGS content; (B) dissolution profiles of binary complex formulation F1, ternary complex formulations (F2, F3) and formulations F2 and F3 without SBE-β-CD in pH 6.8 phosphate buffer medium.

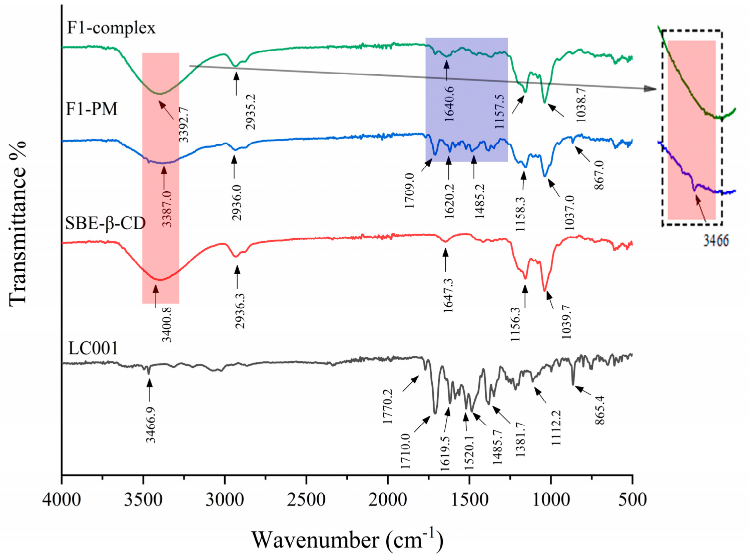

Figure S5. Infrared spectra of pure LC001, SBE-β-CD, and LC001-SBE-β-CD binary systems (F1-PM, F1-complex).

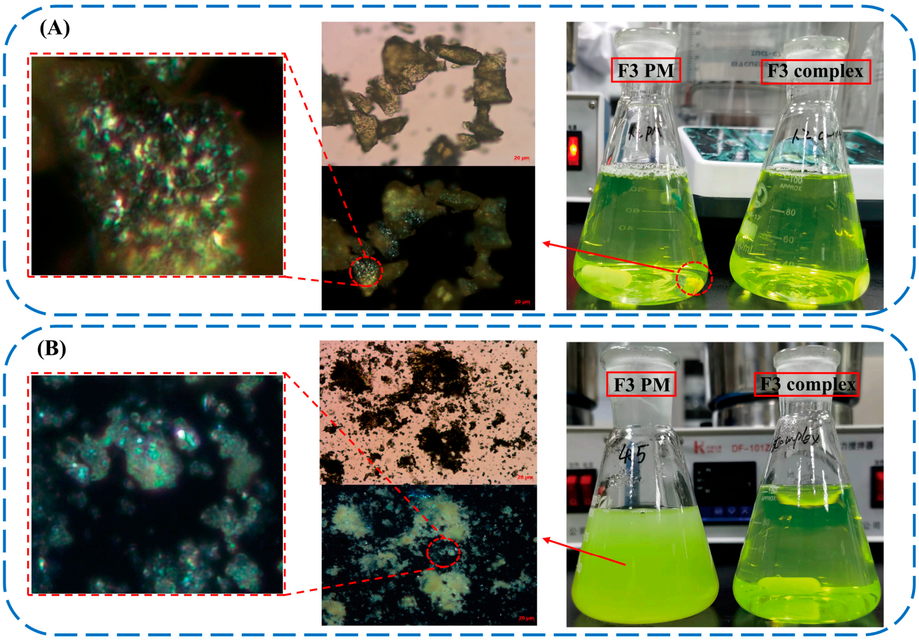

Figure S6. Undissolved solids of ternary complex (F3) and physical mixture (F3 PM) in the dissolution medium: (A) The pH of the dissolution medium changes from 1.2 to 6.8 and (B) from 4.5 to 6.8.

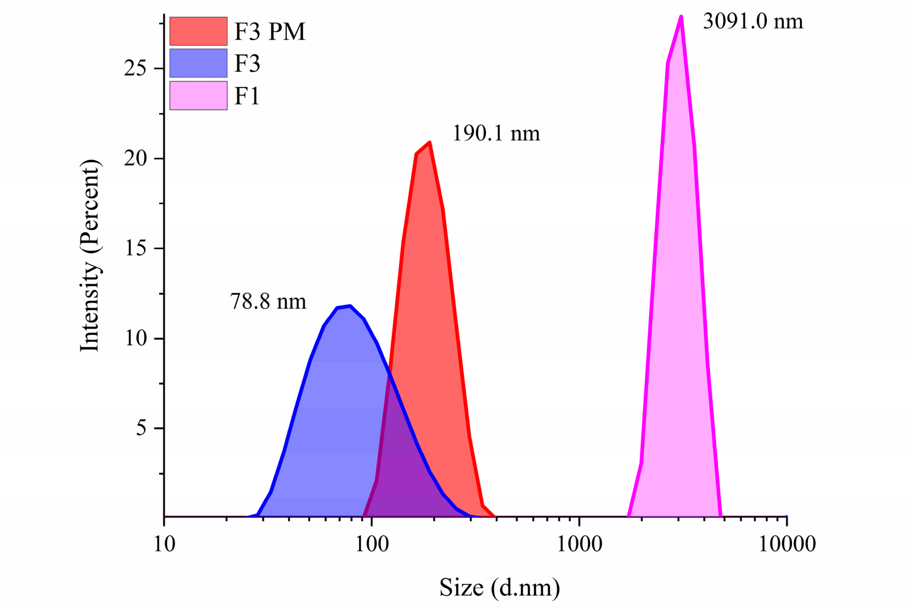

Figure S7. Particle size distribution of binary complex F1, ternary complex F3 and physical mixture F3 PM in pH-shift dissolution media.

Disclaimer/Publisher's Note: The statements, opinions and data contained in all publications are solely those of the individual author(s) and contributor(s) and not of MDPI and/or the editor(s). MDPI and/or the editor(s) disclaim responsibility for any injury to people or property resulting from any ideas, methods, instructions or products referred to in the content.
